# Inappropriate proton pump inhibitor lansoprazole prescription in older adults hospitalized in long-term care unit

**DOI:** 10.1007/s11845-022-03207-3

**Published:** 2022-11-04

**Authors:** Nadia Ladjouzi, Ahmed Romdhani, Georges Zouloumis, Joël Schlatter

**Affiliations:** 1 Long-Term Care Department, Hospital of Paul Doumer, Assistance Publique des Hôpitaux de Paris (AP-HP), Labruyère, France; 2 Pharmacy Department, Hospital of Paul Doumer, Assistance Publique des Hôpitaux de Paris (AP-HP), Labruyère, France

**Keywords:** Inappropriate prescribing, Long-term care, Medication, Older adults, Proton pump inhibitor

## Abstract

**Purpose:**

We evaluated the use of the PPI treatment by physicians in older adults hospitalized in a long-term care unit.

**Methods:**

We included 40 patients aged 65 years or older with a lansoprazole prescription hospitalized in long-term care unit from January 2018 to January 2022. Patient characteristics, gastroduodenal history, dose of lansoprazole, indication, days of prescription, and number of medications were collected from electronic patient records.

**Results:**

The mean age of patients was 84.2 ± 9.3. Patients were taking between 5 and 24 (mean = 12.7, *SD* = 4.4) medications overall with 15 patients taking low dose of aspirin (75 mg daily) and 8 patients taking an antiplatelet. Most patients (82.5%) received once-daily lansoprazole treatment, 55% of whom took a dose of 15 mg. Five patients were treated with the maximum dose of lansoprazole 30 mg twice daily. Only seven patients had an appropriate indication. The minimum of treatment time was 3 days and the maximum was 1198 days; moreover, 24 patients (60%) were still in treatment.

**Conclusion:**

Few PPI prescriptions had an indication in the patient’s electronic record. Prescriptions were ongoing with no date of discontinuation or re-evaluation.

## Key summary points

**Aim:**

To evaluate the lansoprazole prescription in older adults hospitalized in long-term care unit.

**Findings:**

In our study population of 65 years or more, the prevalence of appropriate prescription was 17.5% with mean of medications at 12.7 (*SD* = 4.4). The treatment time was ranging from 3 to 1198 days with 24 patients (60%) still in treatment.

**Message:**

Regular reassessment and the implementation of PPI prescription recommendations were still not routinely used in clinical practice.

## Introduction

Proton pump inhibitors (PPIs) are among the most commonly used medications in the world [1]. They are often prescribed for older patients in common gastroenterological diseases such as gastro-esophageal reflux disease (GERD), esophagitis, prophylaxis of duodenal, and gastric lesions due to non-steroidal anti-inflammatory drugs in patients at risk, *Helicobacter pylori* eradication and the treatment of duodenal and gastric ulcers [1–3]. If the high efficacy and low toxicity of PPIs are properly documented for the short-term prescriptions, the use of drugs has been associated with several adverse effects including drug interactions, fractures, hypomagnesemia, pneumonia, osteoporosis, chronic renal failure, cyanocobalamin deficiency, and *Clostridium difficile*-associated diarrhea [4, 5]. This concern has greater consequences for the older adults hospitalized in long-term care unit as polypharmacy is widely spread in this population more prone to develop adverse effects associated with PPIs. Indeed, PPIs can lead to drug interactions by suppressing gastric acid secretion and interacting via a drug metabolizing enzyme system associated with cytochrome P450. Then, lansoprazole may interfere with the absorption of drugs where gastric pH is critical to bioavailability such as ketoconazole, itraconazole, and digoxin. Moreover, lansoprazole may increase plasma concentrations of drugs metabolized by CYP3A4 such as tacrolimus, carbamazepine, phenytoin, citalopram, and warfarin [6]. In contrast, concomitant use of lansoprazole with clopidogrel was associated with increased risk of major adverse cardiovascular endpoints [7]. Lansoprazole is also a substrate and inhibitor of P-glycoprotein (P-gp) in vitro able to explain some drug interactions via this protein such as digoxin [6, 8].

Except for severe esophagitis or Barrett’s esophagus, as well as prolonged therapy with nonsteroidal anti-inflammatory drugs (NSAID) or antiplatelet agents in patients at risk of upper gastrointestinal bleeding, the duration of PPI therapy should be short-term only for a period of 2 to 12 weeks with the lowest dose possible in elderly population [1, 9, 10]. If pooled data suggested almost half of PPI prescriptions are inappropriate, several literature reviews reported that 20 to 82% of patients received acid-suppressive therapy without an appropriate indication [11–14]. Therefore, some guidelines promoted gradual deprescription of PPIs when are no longer necessary [15, 16].

Thus, the objective of this study is to evaluate the use of the PPI treatment by physicians in older adults hospitalized in a long-term care unit.

## Material and methods

This retrospective cohort study was conducted at the French Paul Doumer geriatric Hospital. Included patients were 65 years of age or older and admitted to long-term care unit between January 2018 and January 2022. Forty patients under lansoprazole during their hospital stay were included in this study. An independent person extracted the records of patients randomly. Data was collected from electronic patient records by two authors (NL and JS). For the entire cohort, the following variables were extracted: age, sex, Autonomy Gerontology Iso-Resources Groups (AGGIR) scale, body mass index (BMI), albumin, C-reactive protein (CRP), gastroduodenal history, date of admission, dose of lansoprazole patient was taking, indication, days of prescription, number of medications, prescription of NSAID, prescription of low dose of aspirin, and prescription of antiplatelet. The AGGIR scale is an autonomy assessment tool used in France for measuring the independency level of elderly people [17]. This scale covers so-called instrumental dimensions that correspond to relatively complex activities with the dominating cognitive component (cooking, medication use, finances, etc.) as well as dimensions with the dominating physical component (so-called fundamental dimensions that are related to such activities as walking, dressing, toileting, etc.). The scale classified the older adults into homogeneous groups from GIR1 meaning dependency in all daily activities to GIR 6 meaning total functional autonomy.

## Results

Table 1 shows inpatient characteristics. The mean age of patients was relatively high at 84.2 ± 9.3. Majority of patients were classified GIR2 including patients confined to bed or chair with cognitive impairment and requiring care for most activities of daily living. Eleven patients (27.5%) were classified GIR1 including patients confined to bed or chair with impaired cognitive functions and requiring ongoing presence of caregivers. The monitoring weight status of patients showed that 13 patients were underweight with a BMI < 23 and 5 patients were overweight with a BMI > 30. Only 10% of patients had a gastroduodenal history that can explain the PPI prescription at the admission. During hospital stay, patients were taking between 5 and 24 (mean = 12.7, *SD* = 4.4) medications overall with 15 patients taking low dose of aspirin (75 mg daily) and 8 patients taking an antiplatelet. Most patients (82.5%) received once-daily lansoprazole treatment, 55% of whom took a dose of 15 mg (Table 2). Five patients were treated with the maximum dose of lansoprazole 30 mg twice daily. Among the 40 patients included in this study, only 7 patients had an appropriate indication (Table 2). Table 3 provided an overview of lansoprazole durations with a mean of 314.4 days (± 306.5). The minimum treatment period was 3 days and the maximum was 1198 days; moreover, 24 patients (60%) were still in treatment.

**Table 1:** Characteristics of the 40 patients treated with lansoprazole

**Characteristics**	**Mean ± SD (extrema)**
Age (years old)	84.2 ± 9.3 (65–102)
Sex ratio (male/female)	17 (42%)/23 (58%)
AGGIR^a^ scale	2 ± 0.9 (1–4)
Serum albumin (g/L)	36.1 ± 4.4 (22.2–44.8)
BMI^b^ (kg/m^2^)	25.1 ± 7.5 (13.5–58.5)
CRP^c^ (mg/L)	14.2 ± 27.0 (1.4–109.5)
Gastroduodenal history	4 patients (10%)
Number of medications • With low dose of aspirin • With antiplatelet	12.7 ± 4.4 (5–24) 15 patients (37.5%) 8 patients (20%)

^a^*AGGIR* Autonomy Gerontology Iso-Resources Groups

^b^*BMI* Body Mass Index

^c^*CRP* C-reactive Protein

**Table 2:** Lansoprazole treatment according to the marketing authorization

**Lansoprazole regimen**	**Number of patients**
15 mg daily	22 (55%)
30 mg daily	11 (27.5%)
15 mg twice	2 (5%)
30 mg twice	5 (12.5%)
**Indications**	**Number of patients**
Indication not found	33 (82.5%)
Appropriate indication • Uncomplicated symptomatic gastroesophageal reflux • Progressive gastric or duodenal ulcers without *Heliccobacter pylori* infection • Prevention of digestive bleeding risk • Prevention of gastrointestinal bleeding during anticoagulation therapy • Prevention of esophagitis relapse with gastroesophageal reflux or peptic ulcer disease	7 (17.5%) 1 1 1 2 2

**Table 3:** Duration of lansoprazole treatment per patient

**Patient**	**Days**	**Date of admission**	**Patient**	**Days**	**Date of admission**
1	> 330 (no end)	08/10/2021	21	> 915 (no end)	03/07/2018
2	> 134 (no end)	02/22/2022	22	50	12/01/2020
3	10	07/28/2020	23	159	11/24/2021
4	168	08/12/2021	24	613	03/02/2018
5	> 586 (no end)	11/27/2020	25	> 1198 (no end)	09/22/2021
6	39	08/06/2021	26	> 91 (no end)	04/06/2022
7	147	11/28/2019	27	> 1193 (no end)	05/02/2018
8	> 125 (no end)	03/03/2022	28	639	07/15/2020
9	> 330 (no end)	08/10/2021	29	311	08/20/2021
10	3	02/11/2021	30	> 256 (no end)	07/23/2020
11	> 110 (no end)	03/18/2022	31	> 195 (no end)	03/28/2017
12	316	01/18/2021	32	> 627 (no end)	07/29/2020
13	10	01/26/2021	33	> 109 (no end)	03/18/2022
14	> 274 (no end)	10/05/2021	34	> 126 (no end)	02/28/2022
15	184	09/03/2018	35	47	07/15/2020
16	75	07/01/2019	36	> 607 (no end)	11/05/2020
17	> 420 (no end)	04/08/2021	373	> 378 (no end)	06/16/2021
18	> 178 (no end)	12/31/2020	383	> 531 (no end)	05/02/2019
19	> 735 (no end)	07/01/2020	39	> 211 (no end)	12/07/2021
20	> 138 (no end)	03/18/2022	40	7	09/06/2021

Mean (days) ± SD, 314.4 ± 306.5 (mini, 3; maxi, 1198); number of patients with no discontinuation, 24

## Discussion

In our study, 82.5% of patients received the PPI lansoprazole treatment without indication in the electronic patient record. This methodology of data compilation represented a limitation of this study, in addition to the relatively modest number of patients included. However, this restriction was supported by almost all literature and was specified in a recent *Cochrane* review that estimated the use of PPI without indication between 20 and 82% of people worldwide [11]. The mean age of the patients in our study was relatively high over 80 years old and patients were polymedicated up to 24 medications. For this purpose, the American Geriatrics Society (AGS) Beers Criteria^®^ included the PPI therapy in its explicit list of potentially inappropriate medication use in older adults, thus inciting physicians to deprescribe PPIs in this population [18]. In addition, the AGS recommended that the use of long-term PPI in the absence of a strong indication should not exceed 8 weeks because of risk of adverse effects (American Geriatrics Society 2015 Updated Beers Criteria for Potentially Inappropriate Medication Use in Older Adults) [19]. What was noteworthy here was that the durations of lansoprazole prescription were widely ranged from 3 days to over 3 years, with treatment continuation in 60% of inpatients hospitalized in long-term care unit. In this iatrogenic risk population, our study proved wrong the hypothesis that hospitalization is an opportunity for regular re-evaluation of PPI treatment. For deprescribing PPIs in older adults, several studies proposed various strategies including actions from physicians or pharmacists [20]. Medication reconciliation at admission is an example of opportunity to check the indication and duration for PPI treatment after verifying the prescriptions of the family physicians, others prescribers, and pharmacist. In our long-term care unit, patients are hospitalized for long time up to few years yielding unlikely the medication reconciliation. To incite physicians to reevaluate PPI therapy, computerized order entry alerts by pharmacists should encourage deprescribing inappropriate drugs.

Retrospective design of this study has limitations. Only information documented in the electronic medical record was exploitable. The absence of appropriate indication of PPI was not mentioned in the electronic medical record.

## Conclusion

This study confirmed that PPI use in older adults was frequent, with only few PPI prescriptions having an indication in the patient’s electronic record. Moreover, prescriptions of lansoprazole were ongoing with no date of discontinuation or re-evaluation. These findings are consistent with the literature, which reported challenges to ensure that PPI prescriptions are in consistence with the recommendations. It is essential to raise physicians’ awareness of PPI prescription recommendations and the need for regular reassessment, but also of the significant side effects that may occur.
